# Social determinants of overweight and obesity in the mother-child binomial: evidences from Mexico

**DOI:** 10.1186/s13690-020-00422-1

**Published:** 2020-05-11

**Authors:** Armando Arredondo, Christian Torres, Emanuel Orozco, Oscar Resendiz

**Affiliations:** National Institute of Public Health, Av. Universidad 655, CP 61100 Cuernavaca, Mor Mexico

**Keywords:** Maternal obesity, Child obesity, Socioeconomic determinants, Dietary patterns, Food security

## Abstract

**Background:**

To analyze the influence of socioeconomic determinants on the development of overweight and obesity in the mother-child binomial.

**Methods:**

This is a study based on a prospective cohort of the mother-child binomial. Using STATA software, the association between the mothers’ body mass index and the nutritional status of minors was analyzed using a logistic regression model with socioeconomic and demographic variables.

**Results:**

The combined prevalence of overweight and obesity in cohort mothers was 53.2%. A statistically significant association was found between the overweight mothers and minors with possible risk of overweight (p 0.001) and with overweight (p 0.001). The logistic regression model was adjusted by age and marital status and linked maternal overweight and obesity with the following variables: severe food insecurity (RR 1.17, CI 0.04–0.31), having a health problem (RR 1.5, CI 0.86–2.05), income (RR 1.79, CI .49–1.30), smoking (RR 1.1, CI 0.80–1.37) and dietary pattern (RR 1.5, CI 0.38–0.87).

**Conclusions:**

The study highlights the importance of paying attention to risk factors starting at the gestational stage, since at this time the mother’s nutritional status has an influence on the offspring’s growth and development. Evidences exist of an association between intergenerational transmission of obesity and socioeconomic aspects of the mother. These evidences must be considered in the revision and adjustments to health system interventions for the prevention of obesity in the mother-child binomial.

## Background

Overweight and obesity are the fastest growing public health problems in developing countries and Mexico is a middle income country with rapid epidemiological and nutritional transitions [1, 2]. According to the 2012 and 2016 National Health and Nutrition Surveys (ENSANUT), the prevalence of these conditions has increased in women of reproductive ages, from 71 to 75%, respectively [3, 4].

Today there is interest in the specific life periods during which diverse environmental exposures induce physiological and anatomical changes, and these in turn can lead to health risk factors. One of the critical periods for women is gestation, where specially their pregestational and gestational body mass indexes (BMI) have an influence on the offsprings’ nutritional status [5, 6].

The associations between nutritional status in early childhood and future risk of chronic diseases have been well studied; these associations may be linked to genetic or environmental factors during pregnancy [7–9]. The evidence has also shown that the mothers’ nutritional status [10–12], has an impact on the offspring’s nutritional status.

Overweight and obesity are multifactorial diseases and socioeconomic factors have become part of the explanation of these conditions. There is evidence that sociodemographic aspects, health-related beliefs and socioeconomic status have an influence on the selection of cheap foods that are high in calories, as well as having less access to healthcare and to healthy foods [11–14]. These factors, together with the demographic, epidemiologic and nutritional transitions, are the main causes of the increase in the prevalence of overweight and obesity.

This investigation was derived from the NUTTSEA [15, 16] cohort, which began in 2017. The sample size was determined by the number of women who sought prenatal care, since week 24 of the gestational period, at a regional referral hospital in the state of Morelos, Mexico. The study’s objective was to analyze the influence of socioeconomic factors related to health disparities, on risks of chronic diseases for mothers and children.

This document shows the findings of the second stage of the NUTTSEA cohort, in order to analyze the influence of socioeconomic determinants on the development of maternal overweight and obesity, and to evaluate these conditions in view of the association of overweight and obesity in their children.

## Methods

This study was based on a cohort type design that was also prospective, analytical and explanatory. The study population consists of the mothers who belong to the NUTTSEA cohort and their children [15, 16]. The universe of study included all pregnant women who requested services for prenatal care during the first semester of 2017, at a regional hospital for uninsured population. The study population was determined by a simple random sampling with statistical power of 90% in the sample size and confidence level of 95%. Pregnant women were included who fulfilled the following criteria: having 24 weeks of gestation or more, living in municipalities near the referral hospital (within a 10 km radius), being over 18 years old and not having plans to move in the short/medium term. We excluded all women who were less than 18 years old, who reported alcohol or tobacco consumption during pregnancy, and who had a history of a disease that could block their inclusion in the study (mental health, amputation, sexually transmitted diseases, blood group incompatibility, etc.).

The data used in the present study were collected by means of quantitative questionnaires directed to the participating mothers. The interviewers who were responsible for the collection of information have training in nutrition and were trained and standardized in anthropometry of adults and children under 2 years of age.

### Socioeconomic - demographic data

A questionnaire was applied with 19 multiple choice questions pertaining to geographic data, as well as data on location, contact, age, marital status, religion, occupation, household income and membership in social programs.

### Diet

Dietary information was obtained by means of a food frequency questionnaire with 103 foods divided into groups and including information on food consumption during the 7 days prior to the application of the questionnaire. In order to obtain dietary patterns, each food was analyzed to determine its composition (kcal, carbohydrates, proteins, lipids, grams and fiber), using the Mexican system of equivalents. 25 food groups were obtained according to their nutritional characteristics.

### Food security

In order to determine the food security indicators, the Latin American Food Security Scale [17] was used. In this scale there are key questions that people face during a certain time period in their homes, pertaining to food availability, access, use and stability.

### Anthropometry

The mothers’ anthropometric weight-height measurements were taken in order to determine the BMI (kg/m^2^). That indicator, was then classified into low weight (< 18.5), normal weight (18.5–24.9), overweight (25–29.9) and obesity (> 30).

The minors’ nutritional status was evaluated by means of three indicators which are: length by age (L/A), weight by age (W/A) and weight by length (W/L). The z score was figured by age and sex, for the whole study population, according to the World Health Organization’s (WHO) reference pattern, and was classified as: malnutrition (severe, moderate and mild), normal, possible risk of overweight (PROW), overweight and obesity. The minors were measured during the first year of their lives. For this purpose, a tray scale was used, parents were asked to remove the child’s clothes, only leaving on a clean diaper. An infantometer was used for the length measurement and with the help of the mother, the anthropometrist made the measurement. The Antro Plus software was used to obtain the diagnosis of the three indicators measured in the minors.

### Data processing and analysis

The categorical and continuous data were presented as percentages and means, respectively. A bivariate analysis was carried out to know the relation between the mothers’ gestational and post-pregnancy nutritional status and the minors’ nutritional status. A chi2 test was done and the *p* < 0.05 value was considered as statistically significant, with confidence intervals of 95%.

The logistic regression model estimated the odds ratio of being overweight and obese and confidence intervals were 95%. Values that were considered to be statistically significant were equal to or under 0.05, 0.01 and 0.001. Maternal BMI was the control variable, categorized as normal/overweight+obesity, adjusting for variables like age, marital status, occupation, family income and total income, educational level, tobacco consumption, alcoholism, municipality of residence and food safety.

To determine dietary patterns, a factor analysis by principal components was carried out with orthogonal rotation (1.6 varimax), which identified the patterns depending on the factor points. Factor loading (> 0.3) showed the importance of a food or food group in a pattern’s definition. Factor loading, variance (eigenvalues > 1.6) for each factor and the interpretability of each component were the elements considered in order to decide the number of extracted factors.

## Results

The findings of the NUTTSEA cohort show data on 396 women, 1 year after the first data collection. These results are divided into three spheres: 1) socioeconomic, which includes economic and demographic population variables; 2) health, which includes health and nutrition status of the mother-child binomial, and 3) food, food security and diet.

### Socioeconomic-demographic characteristics of women in the NUTTSEA cohort

We found that the mean age was 26 years, 63.2% of women lived in common-law marriage, 52.3% had junior high school as their last year of schooling, 80.5% were housewives and only 8.6% had paid work; monthly income from paid work was 91 U. S dlls. In 88.7% of cases, the head of household is the interviewee’s partner and of these, 89% had a paid job with a monthly income of 146 dlls.

With respect to household income in dollars, 14.7% reported having a monthly income of 119 dlls coming from another family member, 9.9% said they received an income of 30 dlls from a social program to which they belonged, and the total household income was calculated as being 207 dlls. 18.2% of the interviewees reported belonging to a social program, being PROSPERA the one that was mentioned the most. With respect to health insurance, 97.8% had the *Seguro Popular*.

### Health

Anthropometric measurements taken after pregnancy show that the mean weight was 59.7 kg (SD 12.7 kg), height was 1.53 m (SD 0.5 m) and BMI was 25.2 (SD 5.1), this last one being three points under the one reported during pregnancy, which was 28 (SD 4.7); however, during pregnancy, the gestational BMI was taken as the cohort point.

Using the BMI (Table 1) as an indicator of the women’s nutritional status, we see that 6.9% of women have low body weight, 39.8% have normal weight, 32.9% are overweight and 20.3% are obese. When comparing to the nutritional status they had during pregnancy, 18.1% had low weight, 32% had normal weight, 31.1% were overweight and 18.6% were obese; thus, we can see an increase in overweight and obesity in the post-pregnant women.

**Table 1:** Anthropometric characteristics of cohort NUTTSEA breasts

	Category	Media ± Standar Deviation, Average 2017^b^	CI 95%	Media, Standar Deviation, Average 2018^a^	CI 95%
Weight	Kg	66.9 ± 12.1	65.37–68.53	59.7 ± 12.7	58.08–61.38
Size	M	1.54 ± 0.5	1.53–1.55	1.53 ± 0.5	1.53–1.54
Body mass index (BMI)	Ms/kg^2^	27.9 ± 4.8	27.50–28.45	25.2 ± 5.1	24.55–25.87
Nutritional diagnosis according to BMI	Under weight	18.1	0.13–0.23	6.9	0.04–0.11
	Normal weight	32	0.26–0.38	39.8	0.33–0.46
	Overweight	31.1	0.25–0.37	32.9	0.27–0.39
	Obesity	18.6	0.14–0.24	20.3	0.15–0.26

^a^ Anthropometry taken 1 year after its first intervention

^b^ Nutrition status based on gestational body mass index. Anthropometry taken from the 24th week of gestation

With respect to minors, a first evaluation was carried out according to the following indicators: weight/age (W/A), length/age (L/A) and weight/length (W/L); this last one was used as a reference point in the nutritional diagnosis (Table 3). 57.5% were boys and 42.5% were girls; the mean reported age in months was 7.35 ± 2.08, for both sexes. Regarding the minors’ anthropometric data, we found that mean weight was 7.74 ± 1.07 kg and mean length was 67.30 ± 5.42, for both sexes.

With respect to W/L, this indicator was taken as a diagnostic criterion for the minors’ nutritional status; 14.72% had mild malnutrition, 3.03% had moderate malnutrition, 0.87% had severe malnutrition, 67.53% had a normal nutritional status, 7.79% were in PROW (Possible Risk of Overweight), 5.63% were overweight and 0.43% had been diagnosed as being obese (Table 2).

**Table 2:** Anthropometric characteristics of children from the NUTTSEA cohort

	Category	Media ± Standar Deviation, Average 2018	CI 95%
Age	Months completed	7.35 ± 2.08	7.08–7.62
	Boys	7.3 ± 2.08	6.94–7.66
	Girls	7.4 ± 2.08	7.01–7.84
Sex	Boys	57.5%	0.51–0.63
	Girls	42.5%	0.36–0.48
Weight	Kg	7.74 ± 1.07	7.60–7.88
Length	Cm	67.30 ± 5.42	66.60–68.00
Nutrition satus (Weight / Age)	Moderate under weight	4.76	0.02–0.08
	Mild under weight	22.08	0.17–0.27
	Normal	64.5	0.58–0.70
	PROW^a^	6.93	0.04–0.11
	Overweight	1.73	0.00–0.04
Nutrition status (Lenght / Age)	Severe low length	2.16	0.00–0.05
	Moderate low length	6.49	0.03–0.10
	Mild low length	24.68	0.19–0.30
	Normal	66.67	0.60–0.72
Nutrition status (Weight / Length)^b^	Severe malnutrition	0.87	0.00–0.03
	Moderate malnutrition	3.03	0.01–0.06
	Mild malnutrition	14.72	0.10–0.19
	Normal	67.53	0.61–0.73
	PROW^a^	7.79	0.04–0.12
	Overweight	5.63	0.03–0.09
	Obesity	0.43	0.00–0.03

^a^Possible risk of overweight; It is a trend towards the scoring line z 2 what is a definite risk

^b^Indicator taken to diagnose the nutritional status of the children evaluated

### Food, food security and diet

ELCSA findings show that 46.3% had food security, 38.1% had mild food insecurity, 10.8% had moderate food insecurity and 4.7% had severe food insecurity. That is, 53.6% suffered from some degree of food insecurity at home. We find evidence that food security is a public health problem, since more than half of our population saw their food limited due to lack of economic resources.

With respect to the dietary analysis, three main components were determined that explain 27% of total variance in consumption. Loading factors (> 0.3) for the three identified patterns are shown in Table 3; a pattern was created, based on three food groups. The pattern of industrialized foods and fats was characterized by consumption of soups (0.30), processed meats (0.35), fats (0.36) and snacks (0.37) and showed a negative load for plain water (0.39). The Western pattern was characterized by consumption of fruits (0.38), sugared cereals (0.30), products derived from corn (0.32) and high fat milk products (0.31), and the pattern with a high sugar factor was characterized by desserts (0.3), sweeteners (0.4) and coffee with added sugar (0.5).

**Table 3:** Characterization of diet patterns by factor analysis in relation to the main components of diet

	Processed foods	Occidental	High in sugar
Vegetables	**–**	**–**	**–**
Fruits high glycemic index	**–**	**0.3842**	**–**
Fruits	**–**	**–**	**–**
Low-fat cereals	**–**	**–**	**–**
Cereals with sugar	**–**	**0.3035**	**–**
Desserts	**–**	**–**	**0.3611**
White bread and flours	**–**	**–**	**–**
Soups	**0.3000**	**–**	**–**
Corn	**–**	**0.3227**	**–**
Red meats	**–**	**–**	**–**
Chicken	**–**	**–**	**–**
Fish and seafood	**–**	**–**	**–**
Processed meats	**0.3588**	**–**	**–**
Legumes	**–**	**–**	**–**
Egg	**–**	**–**	**–**
Liquid dairy	**–**	**–**	**–**
High-fat dairy	**–**	**0.3182**	**–**
Oils	**–**	**–**	**–**
Fats	**0.3608**	**–**	**–**
Other cereals	**–**	**–**	**–**
Snack	**0.3705**	**–**	**–**
Simple water	**−0.3980**	**–**	**–**
Sugary drinks	**–**	**–**	**–**
Sweeteners	**–**	**–**	**0.4860**
Coffee	**–**	**–**	**0.5222**

The diet analysis clearly shows the high consumption of processed and energetically dense foods, which could have been incorporated into the family diet due to their low cost and easy access and availability.

### Bivariate analysis, relation between the mother’s BMI and the minor’s nutritional status

The results of the bivariate analysis for the association between the mothers’ post-pregnancy BMI and the minors’ nutritional status were statistically significant (p.0.05). An association was found between mothers with low weight and minors with moderate malnutrition (p 0.05); mothers with normal weight were linked to a normal nutritional status in minors (p 0.01); mothers who were overweight were associated with PROW (p 0.00) and with overweight (p 0.00) in minors and mothers who were obese were associated with an obesity nutritional status in minors (p 0.00).

### Logistic regression analysis of the socioeconomic variables, including the mother’s nutritional status

The results of the logistic regression model used to determine the association between overweight and obesity in women and socioeconomic variables, are shown in Table 4. Women who experience severe food insecurity (p 0.01, CI 0.00–0.31) have 1.17 higher risk of being overweight and obese, than those who have food security. The findings also showed that not belonging to a social program (p .02, CI 0.01–0.68) leads to 1.87 times greater possibilities of suffering the studied conditions, compared to those who belong to a program. Having a health problem such as hypertension or diabetes (p 0.05, CI 0.86–2.05) results in having 1.5 times higher possibilities of being overweight or obese, compared to those who have no pathology. Being a smoker (p 0.05, CI 0.80–1.37) was positively associated with the above-mentioned conditions and there is a 1.1 higher risk of being overweight or obese than those who did not report being smokers. Consumption of the dietary pattern of processed foods and fat (p 0.001, CI 0.38–0.87), characterized by industrialized soups, snacks and processed meats, led to a 1.5 times risk increase for BMI, compared to those who consumed Western patterns and those that were high in sugar. Finally, although it is not significant, there is a tendency towards overweight and obesity in women who are in the second tertile of income from head of household (p 0.01, CI 0.49–1.32).

**Table 4:** Main results of the logistic regression regarding the determinants of overweight and maternal obesity

Overweight / Obesity	Prevalence %	Odds Ratio (*)	95% CI	***P*** Value
Age				
Tercil 1	36.8	–	–	–
Tercil 2	33.3	2.928513	0.47.18.0	–
Tercil 3	29.8	.9201129	0.11–7.29 s	–
Marital status				
Single	7.7	–	–	–
Married	29	4.120726	0.18–90.86	–
Free union	63.2	3.401186	0.17–65.97	–
Employment of Family Chief				
Remunerated	2.1	–	–	–
Unpaid	89.1	.0757522	0.00–19.45	–
Unemployed	8.66	.6922784	0.00–21.45	–
Family Chief Income				
Tercil 1	36.6	–	–	–
Tercil 2	33.4	.7917301	0.49–1.32	**
Tercil 3	29.9	.2105857	0.01–2.71	–
Total income				
Tercil 1	36.8	–	–	–
Tercil 2	30.7	9.405535	0.82–10.75	–
Tercil 3	32.4	10.36989	0.45–23.87	–
Mother occupation				
Remunerated	19	–	–	–
Unpaid	80.9	3.622143	0.36–35.69	–
Level of education				
Low	67.9	–	–	–
High	32	1.163893	0.21–6.33	–
*P. Processed foods*	100	1.5816062	0.38–0.87	*
*P. Occidental*	100	.7709485	0.47–1.25	–
*P. High in sugar*	100	1.157448	0.64–2.07	–
Smokes				
No	95.6	–	–	–
Yes	4.3	1.1299077	0.80–1.37	***
Alcohol				
No	72.2	–	–	–
Yes	27.7	1.101313	0.25–4.80	–
Municipality				
Temixco	52.8	–	–	–
E. Zapata	21.2	.4058108	0.05–2.78	–
Xochitepec	25.9	1.595282	0.30–8.46	–
Social program				
Yes	18.1	–	–	–
No	81.8	1.0877091	0.01–0.68	**
Food safety				
Safety	45.8	–	–	–
Mild insecurity	38.9	2.609584	0.57–11.88	–
Moderate insecurity	10.8	.8550086	0.11–6.33	–
Severe insecurity	4.3	1.1700472	0.00–0.31	**
Current health problem				
No	88.3	–	–	–
Yes	11.6	1.572707	0.86–2.05	***
Pregnancy health problem				
No	70.5	–	–	–
Yes	29.4	.7714542	0.12–4.87	–

< 0.001* < 0.01** < 0.05*** Odds ratio adjusted by age and marital status

## Discussion

The NUTTSEA cohort is a study that analyzes the interaction and influence of socioeconomic determinants as risk factors for overweight and obesity in mothers, and how this in turn affects the nutritional status of the offspring. Our results show evidence of a significant positive association between intergenerational transmission of obesity and the mother’s socioeconomic aspects.

An excessive weight increase during pregnancy has been associated with overweight and obesity in post-partum women [18, 19]. In the NUTTSEA cohort we were able to observe that the combined prevalence of both conditions during pregnancy was 49.7% and 1 year after the survey it was 53.2%. Although the association was not statistically significant, the prevalence increased by almost four percentage points.

The joint prevalence of overweight and obesity in minors in the NUTTSEA cohort was 6% and for PROW it was 7.9%, which suggests a future increase in the prevalence of both conditions. This information may be compared to data from the 2016 ENSANUT survey, whose prevalence was 12.3% [20] and data from the 2015 National Survey of Boys, Girls and Women, which reported a prevalence of 5.2% [21]. These national surveys considered 5 year old minors as a reference and our study only considered minors who were 1 year old. Today, no data exist on overweight and obesity in breastfeeding babies.

Our results show that excess weight in the mother is significantly associated with PROW and overweight (p 0.05) in minors. We were able to observe that overweight in mothers was associated with PROW (p 0.00) and with overweight (p 0.00) in a statistically significant way. Other studies report similar findings, where overweight and obesity in parents lead to the development of these conditions in minors [9–11], and relate the appearance of chronic and metabolic diseases in adult children of obese parents [7, 22, 23]. With respect to the socioeconomic determinants, we were able to observe that severe food insecurity was significantly associated (p 0.02) with an increase in BMI in mothers. These results agree with those from other investigations which relate the development of overweight or obesity with some degree of food insecurity, since this is considered to be a factor predisposing to access and availability of dense energy foods that are high in saturated fats, as well as processed foods [24, 25].

We explored the association and influence on nutritional status of not belonging to a social program, specifically its effect on overweight and obesity, and found a positive association (p 0.02, CI 0.01–0.68). Our results agree with some studies showing that these programs provide orientation and education in matters of nutrition, as well as monetary incentives to improve food intake, or else participants receive food supplements [26, 27].

With respect to lifestyles, we found that consumption of a dietary pattern of processed food, characterized by industrialized soups, snacks and processed meats, was related to the studied conditions (p 0.001, CI 0.38–0.87). Other studies of dietary patterns associate this type of pattern with weight gain, abdominal obesity or diabetes [28–30]. Another studied lifestyle was that of tobacco smokers, where we found a significant connection between smoking and BMI in women (p 0.05, CI 0.80–1.37). Other studies also talk about weight gain with cigarette consumption and this is considered to be a risk factor for chronic diseases [31]. We found evidence that dietary patterns characterized by processed foods are influenced by family economics, access and availability. It has also been observed that the most vulnerable households are more prone to consumption of this pattern since the densely energetic foods are cheaper.

The study’s limitations are that some women who were renting moved to a different address and also changed their phone numbers; this prevented us from being able to contact them, thus representing a loss in the sample. In some cases, there was a death of a minor, for which the mothers were not surveyed. With respect to the economic indicators, it is worth mentioning that we only reported the perception of income since we do not have an economic index to estimate the families’ real incomes.

## Conclusion

Our results highlight the importance of paying attention to risk factors present since the gestational stage, in view of the fact that during this period the mother’s nutritional status has an influence on the offspring’s growth and development, and it is during this stage of early infancy when the health conditions of the adult stage are defined. This investigation shows evidence of a positive and significant association between the intergenerational transmission of overweight and obesity and the mother’s socioeconomic aspects. These evidences could be considered in the design and implementation of interventions and/or public health programs, as strategies for the prevention of overweight and obesity in the mother-child binomial.

## Data Availability

Applicable.

## Abbreviations

ENSANUT: National Health and Nutrition Surveys (Abbreviation in Spanish)
BMI: Body mass index
NUTTSEA: Name of the cohort
L/A: Length by age
W/A: Weight by age
WHO: World Health Organization
PROW: Possible risk of overweight
ELCSA: Escala Latinoamericana y del Caribe de Seguridad Alimentaria
