# Correction to: Social determinants of overweight and obesity in the mother-child binomial: evidences from Mexico

**DOI:** 10.1186/s13690-020-00434-x

**Published:** 2020-06-03

**Authors:** Armando Arredondo, Christian Torres, Emanuel Orozco, Oscar Resendiz

**Affiliations:** National Institute of Public Health, Av. Universidad 655, CP 61100 Cuernavaca, Mor Mexico

**Correction to: Arch Public Health (2020) 78: 42**

**https://doi.org/10.1186/s13690-020-00422-1**

Following publication of the original article [1], the authors would like to correct the number of RR in Abstract section and Table 4.

**Table 4:** Results of the logistic regression model on determinants of maternal overweight and obesity

***Overweight/Obesity***	***Prevalence %***	Odds Ratio (*)	***95% CI***	***P Value***
*Age*				
Tertil 1	36.8	-	-	-
Tertil 2	33.3	2.928513	0.47.18.0	-
Tertil 3	29.8	.9201129	0.11–7.29	-
*Marital status*				
Single	7.7	-	-	-
Married	29	4.120726	0.18–90.86	-
Free union	63.2	3.401186	0.17–65.97	-
*Chief Family Employment*				
Remunerated	2.1	-	-	-
Unpaid	89.1	.0757522	0.00–19.45	-
Unemployed	8.66	.6922784	0.00–21.45	-
*Chief Family Income*				
Tertil 1	36.6	-	-	-
Tertil 2	33.4	.7917301	0.49–1.30	**
Tertil 3	29.9	.2105857	0.01–2.71	-
*Total income*				
Tertil 1	36.8	-	-	-
Tertil 2	30.7	9.405535	0.82–10.75	-
Tertil 3	32.4	10.36989	0.45–23.87	-
*Mother occupation*				
Remunerated	19	-	-	-
Unpaid	80.9	3.622143	0.36–35.69	-
*Level of education*				
Low	67.9	-	-	-
High	32	1.163893	0.21–6.33	-
*P. Processed foods*	100	1.5816062	0.38–1.87	*
*P. Occidental*	100	.7709485	0.47–1.25	-
*P. High in sugar*	100	1.157448	0.64–2.07	-
*Smokes*				
No	95.6	-	-	-
Yes	4.3	1.1299077	0.80–1.37	***
*Alcohol*				
No	72.2	-	-	-
Yes	27.7	1.101313	0.25–4.80	-
*Municipality*				
Temixco	52.8	-	-	-
E. Zapata	21.2	.4058108	0.05–2.78	-
Xochitepec	25.9	1.595282	0.30–8.46	-
*Social program*				
Yes	18.1	-	-	-
No	81.8	1.0877091	0.01–0.68	**
*Food safety*				
Safety	45.8	-	-	-
Mild insecurity	38.9	2.609584	0.57–11.88	-
Moderate insecurity	10.8	.8550086	0.11–6.33	-
Severe insecurity	4.3	1.1700472	0.00–1.31	**
*Current health problem*				
No	88.3	-	-	-
Yes	11.6	1.572707	0.86–2.05	***
*Pregnancy health problem*				
No	70.5	-	-	-
Yes	29.4	.7714542	0.12–4.87	-

In Abstract, the sentence currently reads:

‘severe food insecurity (RR 1.17, CI 0.04–0.31), having a health problem (RR 1.5, CI 0.86–2.05), income (RR 1.79, CI .49–1.30), smoking (RR 1.1, CI 0.80–1.37) and dietary pattern (RR 1.5, CI 0.38–0.87)’.

The sentence should read:

‘severe food insecurity (RR 1.17, CI 0.04–1.31), having a health problem (RR 1.5, CI 0.86–2.05), income (RR 0.79, CI .49–1.30), smoking (RR 1.1, CI 0.80–1.37) and dietary pattern (RR 1.5, CI 0.38–1.87)’.

The original article has been corrected.
